# Long-Acting Injectable Antipsychotics—A Review on Formulation and In Vitro Dissolution

**DOI:** 10.3390/pharmaceutics16010028

**Published:** 2023-12-24

**Authors:** Magdalena Markowicz-Piasecka, Marcin Kubisiak, Katarzyna Asendrych-Wicik, Michał Kołodziejczyk, Joanna Grzelińska, Małgorzata Fabijańska, Tomasz Pietrzak

**Affiliations:** 1 Department of Applied Pharmacy, Medical University of Lodz, ul. Muszynskiego 1, 90-151 Lodz, Poland; 2 Liquid Dosage Form Laboratory, Research and Development Department, Polfa Warszawa S.A., Karolkowa 22/24, 01-207 Warsaw, Poland; marcin.kubisiak@polpharma.com (M.K.); k.asendrych-wicik@polfawarszawa.pl (K.A.-W.); jm.grzelinska@gmail.com (J.G.); t.pietrzak@polfawarszawa.pl (T.P.); 3 Department of Pharmaceutical Chemistry, Drug Analysis and Radiopharmacy, Medical University of Lodz, ul. Muszynskiego 1, 90-151 Lodz, Poland; 4 Department of Drug Form Technology, Medical University of Lodz, ul. Muszynskiego 1, 90-151 Lodz, Poland; michal.kolodziejczyk@umed.lodz.pl; 5 Department of Bioinorganic Chemistry, Medical University of Lodz, ul. Muszynskiego 1, 90-151 Lodz, Poland; malgorzata.fabijanska@umed.lodz.pl; 6 Faculty of Chemistry, Warsaw University of Technology, Noakowskiego 3, 00-664 Warsaw, Poland

**Keywords:** neuroleptics, long-acting injectables (LAIs), in vitro drug release, in vitro–in vivo correlation (IVIVC)

## Abstract

Long-acting injectable (LAI) neuroleptics constitute an effective therapeutical alternative for individuals suffering from persistent mental illness. These injectable pharmaceuticals help patients manage their condition better and improve long-term outcomes by preventing relapses and improving compliance. This review aims to analyse the current formulation aspects of LAI neuroleptics, with particular emphasis on analysis of drug release profiles as a critical test to guarantee drug quality and relevant therapeutical activity. While there is no officially approved procedure for depot parenteral drug formulations, various dissolution tests which were developed by LAI manufacturers are described. In vitro dissolution tests also possess a critical function in the estimation of the in vivo performance of a drug formulation. For that reason, thorough inspection of the in vitro–in vivo correlation (IVIVC) is also discussed.

## 1. Introduction

The first mentions of sustained drug release (SR) technology date back to 1938, when the patent of Israel Lipowski was published [1]. His research included coated pellets for the sustained release of a drug. This scientific position can be considered a milestone in the advancement of the coated particle approach to sustained drug delivery which was discovered in 1950s [2]. Currently, pharmaceutical technology and the search for new therapeutics and drug products is in high demand. Sustained drug release at specific sites is clinically favourable for the treatment and management of many diseases, including central nervous system (CNS) diseases, and scientists attempt to design and develop more innovative and efficient approaches for the treatment of multiple disorders [3,4,5]. Simultaneously, appropriate dose, correct application and site targeting are crucial for therapeutic success. Sustained drug release technology provides several benefits and potential compensation contrary to conventional drug formulations. For instance, sustained drug delivery constitutes a way of improving the therapeutic effectiveness of incorporated drug substances by providing their stable concentrations and targeting the pertinent site [6]. In addition, drug plasma levels persist within the therapeutic range for a greater period of time compared to the traditional products. Importantly, sustained drug delivery systems improve the probability for the patient to respond to the healing treatment because these drug formulations reduce the need for frequent application of the drug. On the other hand, the most severe risk associated with the use of SR drugs is prolonged time of occurrence of possible adverse effects with no effective means of reducing the offensive dose [7]. Also, after the treatment is finished, disappearance of the potentially harmful side effects is delayed, compared to short acting formulations [8].

SR systems constitute any drug delivery system that provides the slow release of a drug substance over a prolonged time. The rate at which the drug substance is released from SR products relies upon many elements, with excipients and polymers playing the most crucial role. If the formulation allows for constant drug concentrations in the desired site, it is regarded as a controlled-release system. A special group of SR formulations is drugs for parenteral administration, including injections. Modified release (MR) parenteral pharmaceuticals are available in several delivery systems including microspheres, liposomes, gels, suspensions, implants, and lipophilic solutions [3,5]. Taking into consideration the type of drug release, one can distinguish delayed-release, extended-release (ER) or sustained-release products [9,10]. Long-acting injections serve as a big change in the way pharmaceutics are administered, allowing patients to abandon taking the medicine frequently using a modern drug delivery method and instead go weeks between dosages. As the need for long-acting injectables (LAIs) grows, so does the demand for innovatory drug delivery systems. LAIs have become an important part of the treatment of numerous diseases, including chronic pain, hormonal contraception, central nervous system diseases (bipolar disorder, schizophrenia), bone diseases, cancer, or migraine. On the other hand, the features of LAIs that make these products so advantageous also make formulation and product development a challenge for pharmacists at pharmaceutical companies.

LAIs offer multiple advantages for patients. For instance, these formulations can provide a more consistent drug concentration in the body, which can lead to fewer side effects, improved symptom control and faster healing. LAIs are also easier to administer than other more traditional forms of pharmaceuticals, such as tablets because LAIs are injected once or twice per month. In the case of chronic pain local to specific areas of the body, LAIs allow for improved targeting, higher drug concentration in the area of the disease, and simultaneous lower systemic exposure and subsequent decreased risk of side effects [11]. In the case of the physician’s perspective, LAIs can be a valuable tool in treating chronic diseases because they provide better compliance by making it easier for patients to maintain their treatment regimen. Since LAIs maintain a more stable drug concentration in the body, they can be more effective in preventing relapses [12,13]. Importantly, LAIs also give hope for reducing drug abuse because they must be administered by a healthcare professional. Importantly, LAIs have the potential to reduce healthcare costs due to reduced number of doctor’s visits, and reduced risk of relapse [13].

Therefore, this paper aims to review the formulation aspects of parenteral drugs with modified release putting particular emphasis on LAI antipsychotics. For this purpose, we provide a brief introduction on neuroleptics and describe the current formulation aspects of LAI neuroleptics. The pharmaceutical analysis of available pharmaceutical preparations of neuroleptics is also broadly described. The complicated nature of LAI formulations constrains the use of in vitro release testing as a factor determining the formulation’s action. Unfortunately, the development of in vitro dissolution test approaches that enable us to foresee the in vivo performance of these systems is limited by the need of standard methods [14]. Therefore, this paper provides several examples of in vitro drug release tests, as well as the correlation between in vitro and in vivo (IVIVC) release of some LAI antipsychotics.

## 2. Overview of Neuroleptic Medications

Antipsychotics are commonly prescribed for the treatment of numerous diseases of the CNS. In most cases, they exert tranquillizing effects and are administered to treat psychosis or bipolar disorders. Antipsychotics are generally classified into three groups: first-generation antipsychotics (FGAs, also known as “typical”), second-generation antipsychotics (SGAs, “atypical”) and third-generation antipsychotics (Table 1). The typical neuroleptics are divided according to their chemical structure, while the atypical group is categorized corresponding to pharmacological activity [15].

Generally, this heterogeneous group of drugs is administered in the treatment of multiple neuropsychiatric diseases, including psychosis, attention-deficit hyperactivity disorder (ADHD), behavioural disturbances in dementia, geriatric agitation, depression, eating disorders, personality disorders, insomnia, generalized anxiety disorder, obsessive-compulsive disorder, and post-traumatic stress disorder (PTSD) [16]. Some of these indications are currently considered *off label* and the efficacy of antipsychotics in their treatment is inconclusive. 

Older neuroleptics, which include FGAs, were synthesized and approved in the 1950s, with chlorpromazine being the first approved drug in this class [17]. These drugs were not only used in psychotic disorders, but also in the treatment of acute mania, agitation, bipolar disorder, Tourette syndrome, and hyperactivity. However, the use of chlorpromazine and other classic neuroleptics such as haloperidol was correlated with a high risk of development of adverse side effects. After about 30 years of using FGAs, the SGAs were developed in the 1980s. Clozapine was the first second-generation neuroleptic with minimal or no extra-pyramidal effects [18]. Unfortunately, clozapine was found to induce agranulocytosis [19], which contributed to the restriction in its use or even withdrawal. Clozapine became a prototype for the synthesis of other SGAs such as risperidone, olanzapine, or quetiapine. The examples of the newest SGA antipsychotics include paliperidone (a derivative of risperidone), iloperidone, and lurasidone. These drugs are characterized by a favourable efficacy and safety profile [18]. Table 1 presents the general classification of neuroleptics.

**Table 1:** General classification of antipsychotics [20,21,22].

Typical Antipsychotics (First Generation)			
**Phenothiazines**	**Thioxantenes**	**Butyrophenones**	**Diphenylbutylpiperidine**
**Chlorpromazine**	Chlorprotixene	Haloperidol	Pimozide
**Flufenazine**	Clopentixol	Droperidol	Penfluridol
**Perfenazine**	Flupentixol	Bromperidol	
**Prochlorperazine**	Zuclopenthixol	Benperidol	
**Thioridazine**			
**Trifluoperazine**			
**Mesoridazine**			
**Promazine**			
**Triflupromazine**			
**Promethazine**			
**Levomepromazine**			
**Cyamemazine**			
**Atypical antipsychotics (second generation)**			
**Clozapine,**		Olanzapine,	
**Risperidone**			
**Quetiapine,**		Ziprasidone,	
**Amisulpiride**			
**Asenapine,**		Paliperidone,	
**Iloperidone**			
**Zotepine,**		Sertindole,	
**Lurasidone**			
**Third generation**			
**Aripiprazole**			
**Brexpiprazole**			
**Cariprazine**			

One of the most important distinguishing features of first- and second-generation antipsychotics is their interaction with various receptors determining their mechanism of biological activity. In the case of FGAs, the postsynaptic restriction of dopamine D_2_ receptors in the CNS is the mechanism of action. Typical antipsychotics have to meet certain criteria to act effectively. These include the blockade of D_2_ receptors in striatal and cortical areas, a greater association between D_2_ receptor binding and its potency, and 65% D_2_ receptor occupancy. Unfortunately, the nonspecific localization of dopamine receptors in the CNS is related with the risk of the development of movement disorders and hyperprolactinemia [23,24]. 

On the contrary, atypical antipsychotics transiently occupy D_2_ receptors and rapidly dissociate from them, which allows for normal dopamine neurotransmission. In addition, they block 5-HT_2A_ receptors and activate 5-HT_1A_. Importantly, SGAs possess fewer side effects, and do not induce extrapyramidal adverse effects; however, their safety in elderly populations is debatable [25]. 

The adverse side effects of typical antipsychotics stem from their effects on other receptors, including 5-HT_2A_, α_1_-adrenergic receptors, histaminic H_1_, and muscarinic receptors. Based on the differences in the effects on these receptors, FGAs can be divided into high- and low-potency therapeutics. Fluphenazine, trifluoperazine, haloperidol, pimozide, and perphenazine are high-potency, typical antipsychotics, administered at a dose between one and tens of milligrams. The most frequently occurring side effects are weight gain, sedative effects, and anticholinergic activity. They introduce a high risk of extrapyramidal side effects (dystonia, bradykinesia, rigidity, tremor, and tardive dyskinesia), and hyperprolactinemia. In turn, chlorpromazine and thioridazine are regarded as low-potency antipsychotics and are used in higher doses reaching hundreds of milligrams. These drugs exhibit a high affinity towards histamine and muscarinic receptors with associated greater risk of sedation and anticholinergic effects, but show a lower tendency to develop extrapyramidal side effects [26,27].

The use of SGAs is not linked with extrapyramidal and anticholinergic effects; their activity is, however, not free from other side effects. These include weight gain, type 2 diabetes mellitus (T2DM), metabolic syndrome, drowsiness, sedation, and QTc prolongation. Additionally, the use of clozapine, a potent drug in the treatment of psychotic symptoms and suicidality, is associated with a high risk of agranulocytosis [19].

**Figure 1:**
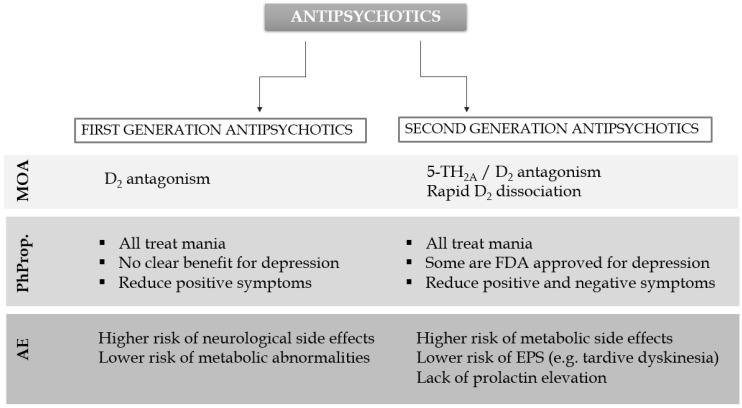
A schematic comparison of basic features of FGA and SGA. MOA—mechanism of action; PhProp—pharmacological properties; EPS—extrapyramidal symptoms; AE—adverse effects.

The toxicity of neuroleptics stems from their interactions with the following receptors: dopamine (extrapyramidal symptoms), α_1_-adrenergic (orthostatic hypotension, reflex tachycardia), muscarinic (anticholinergic symptoms), and histaminic receptors (sedation) [28,29]. Symptoms of neuroleptic overdose can be overcome using diphenhydramine or benzatropine. The most life-threatening symptom associated with the use of antipsychotics is a neuroleptic malignant syndrome (NMS) [27,30], the occurrence of which is mainly associated with first-generation antipsychotics. The clinical manifestation of this dangerous complication includes altered mental status, muscular rigidity, hyperthermia, and autonomic dysfunction [31]. A comparison of the basic properties of antipsychotic FGA and SGA drugs is presented graphically (Figure 1).

Most FGAs are used in oral formulations which are dictated by either sublingual absorption (only asenapine) or in the gastrointestinal tract. The factors affecting the rate and extent of absorption of oral neuroleptics are as follows: drug dissolution rate, drug solubility, susceptibility to enzymatic reactions, gastric pH, and drug–drug and food–drug interactions [32]. Most FGAs are metabolized in the liver; therefore the first-pass effect, which reduces the bioavailability of drugs, must also be taken into account. Cytochrome P450 (CYP) alleles are responsible for the metabolism of 70–90% of FGAs. Changes in CYP enzyme activity, primarily due to drug–drug interactions, can necessitate the adjustment of the dosing regimen of FGAs. For instance, the drugs which inhibit isoforms of CYP (e.g., CYP2D6) can decrease the rate of metabolism of FGAs and therefore increase their concentrations leading to adverse reactions. Conversely, the concentration of neuroleptics can be reduced due to the coadministration of drugs being inducers of CYP enzymes. Other important issues affecting oral FGA concentration are patient-related factors including impaired hepatic function or age-related decline in liver function [32,33]. 

Some FGAs are also available in injectable intramuscular formulations, which are particularly helpful in the treatment of psychotic agitation. Intravenous injections of haloperidol and droperidol are used for the treatment of psychosis, agitation, or delirium in acute medical settings. In turn, haloperidol and fluphenazine are available in the form of long-acting formulations, which are dedicated for individuals who do not respond to the drug dosing regimen. SGAs are available in oral forms, intramuscular injections (aripiprazole), and LAIs (e.g., olanzapine, risperidone, paliperidone, and aripiprazole) [34]. The use of sustained-release neuroleptics, particularly long-acting injectables, will be discussed in detail in the next section.

## 3. Long-Acting Injectable (LAI) Antipsychotics

SR technology is a rapidly growing interdisciplinary science offering unconventional strategies for the systemic delivery of pharmaceuticals into the circulatory system at a fixed rate. Design and development of a predictive and reduplicative drug release rate for a prolonged and established time allow for the achievement of favourable therapeutic reactions, extended efficacy and reduced toxicity [35]. Taking into consideration neuroleptics, one can also think of new formulations ensuring the prolonged release of the active substance; these are known as LAI antipsychotics. The first LAI introduced to the pharmaceutical market was fluphenazine in the form of enanthate and decanoate. These formulations were approved in 1966 in the context of the huge deinstitutionalisation of subjects with serious psychiatric diseases and the subsequent need for effective community-based therapy [36]. Table 2 summarizes FGAs and SGAs available in the form of LAIs. These groups differ in terms of strength in relation to the negative symptoms and the enhancement of cognitive functions. Both groups differ regarding tolerability, toxicity, and the risk for tardive dyskinesia [37].

The use of a modern formulation such as LAIs is associated with a change in the pharmacokinetics of neuroleptics. The first and most important factor is drug absorption from the injection site which is influenced by water solubility of the drug and the properties of the delivery vehicle, as well as patient factors including body weight, subcutaneous fat, and vascularity of the site of injection [32]. In the case of most LAI neuroleptics, a large percentage of the administered drug initially remains in the injection area. Therefore, the absorption rate of the drug is slower than the elimination rate. This phenomenon is known as “flip-flop” kinetics, resulting in the time to steady state being a function of absorption. Simultaneously the concentration of the drug at steady state is a function of its elimination [38]. LAI neuroleptics are absorbed slowly and gradually from the injection site, which contributes to the prolonged concentration of the drug in the bloodstream. After reaching the bloodstream, the pharmacokinetic characterization, e.g., the distribution and elimination parameters of LAI antipsychotics, is the same as after oral dosing. Subcutaneous and intramuscular injection of LAI antipsychotics may contribute to an increase in drug bioavailability due to avoidance of the first-pass metabolism in the liver [32]. As presented by Sheehan et al. [39], the slower absorption rate of LAI drugs contributes to reduced peak (Cmax)-to-trough (Cmin) plasma concentration differences, which can result in fewer side effects and greater tolerability. Generally, peak-to-trough plasma concentrations differ depending on the drug, its dosing and formulation. The greater the fluctuations in peak-to-trough concentrations, the higher the risk of adverse side effects and lower tolerability [39]. The available data suggest that antipsychotic formulations and a dosing regimen resulting in a peak-to-trough fluctuation of two or less is optimal to reach a balance between efficacy and tolerability [39].

Therapy with LAIs should be considered for subjects with recent-onset schizophrenia and those with risk factors for medication non-compliance (such as a history of non-adherence, severe symptoms, comorbid substance use, cognitive impairment, etc.) [36]. They are intended for individuals requiring a long-term antipsychotic therapy lasting at least several months [37]. 

LAI antipsychotic pharmaceutics are usually used every two or four weeks instead of the daily dosing required for oral formulations. LAI neuroleptics were initially developed to improve the compliance of patients with chronic psychosis; however, it was found that they possess more advantages which will also be discussed herein [40,41]. For instance, LAI antipsychotics ensure that the drug is delivered for a specified period of time, which eliminates questions regarding the proper and regular administration of a drug. One should also remember about problems of individuals suffering from schizophrenia, particularly impaired thinking or memory difficulties, which makes it difficult to take medications every day. Therefore, LAIs allow an increase in the comfort of use [40,42]. The current clinical guidelines in psychiatry indicate LAI therapy as a preferred therapeutic strategy for subjects with a significant history of nonadherence [43]. The use of LAI antipsychotics also enables easy detection of cases of nonadherence (e.g., not showing up for the injection), whereas nonadherence to oral medications is frequently undetected until a major problem occurs [44]. In addition, LAI antipsychotics reduce the risk of unintentional or deliberate overdose and provide clarity of adherence [45]. Finally, LAIs provide a few advantages concerning pharmacokinetic characteristics, including more consistent bioavailability [46], a greater correlation between dosage and plasma concentrations [47], better subject outcomes and satisfaction [48], and lower relapse rates than oral therapy [49]. LAIs promote the use of the lowest effective dose principle, reducing the prevalence of adverse side effects [50].

**Figure 2:**
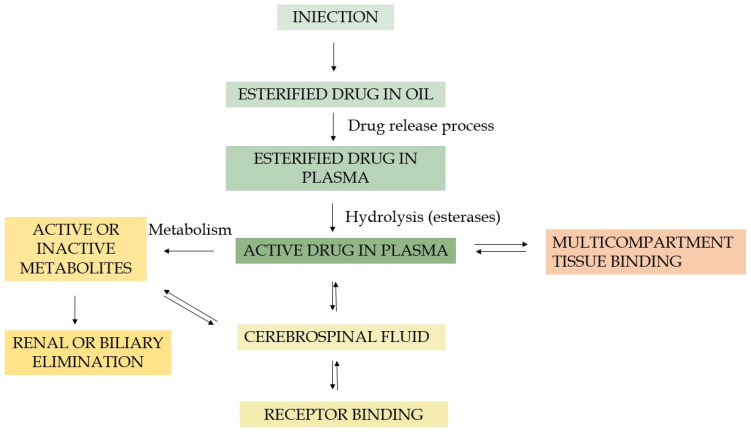
Disposition of long acting injectable (LAI) antipsychotics.

The FGA LAI drugs (Table 2) are formed in an esterification reaction between a terminal alcohol group and carboxylic acid (vide infra). The highly hydrophobic aliphatic esters (e.g., decanoate and palmitate) are usually mixed in vegetable oils. Once injected into a muscle, these products form a reservoir of active ingredient that is slowly dissolved in the surrounding blood where endogenous esterases hydrolyze the esters and release the active compound (Figure 2). These formulations enable the obtaining of a favourable pharmacokinetic profile, and sustained release of a drug. For instance, after intramuscular gluteal administration, peak plasma concentrations of fluphenazine decanoate appear within 24 h, and the apparent half-life is ~7–10 days. Fluphenazine is metabolized (fluphenazine sulfoxide, 7-hydroxy fluphenazine, and fluphenazine N-oxide), undergoing “first-pass” metabolism by the liver, and is excreted in both urine and faeces [32]. In turn, haloperidol decanoate is slowly released into the bloodstream and immediately hydrolysed, resulting in active haloperidol. Peak plasma concentrations of the drug occur between 3 and 9 days, with an elimination half-life of ca. 3 weeks [32]. 

In the case of second-generation antipsychotics, the issue of sustained release is much more complex, but it is generally a drug form that is sparingly soluble when administered intramuscularly and releases the active ingredient slowly. For instance, aripiprazole monohydrate is used in a form of lyophilized powder which is suspended in sterile water before the injection and is gradually absorbed into the blood stream because of its low solubility [51]. The median time to peak plasma concentration occurs within 4 days following multiple deltoid injections and 5–7 days following multiple gluteal injections. The apparent half-life is within the range 29.9–46.5 days [32]. Another example is aripiprazole lauroxil, which slowly dissolves into the systemic circulation, where it is first converted to N-hydroxymethyl aripiprazole and lauric acid. The first compounds are then quickly transformed into aripiprazole. Peak plasma concentrations occur approximately 41 days after a single administration. The elimination half-life of aripiprazole lauroxil is approximately 53.9–57.2 days [32].

**Table 2:** First-, and second-generation antipsychotics available as long-acting injectable medications [32,52,53].

Drug	Available Formulation	Maintainance Dose (mg) and Injection Interval
First-generation antipsychotics		
Haloperidol decanoate	50 and 100 mg/mL solution for injection	50–200 mg every 3–4 weeks
Fluphenazine decanoate	100 mg/mL solution for injection	12.5–50 mg every 2–3 weeks
Flupenthixol decanoate	20 mg/mL solution for injection	50–300 mg every 2–4 weeks
Zuclopenthixol decanoate	200 mg/mL solution for injection	200–500 mg every 1–4 weeks
Second-generation antipsychotics		
Aripiprazole monohydrate	300, 400 mg vials, prefilled syringes	400 mg once/month
Aripiprazole lauroxil	441, 662, 882 mg prefilled syringes	441–882 mg once/month
Olanzapine pamoate	210, 300, 405 mg vials	150–300 mg every 2 weeks or 300–405 mg every 4 weeks
Paliperidone palmitate	39, 78, 117, 156 or 234 mg prefilled syringes	39–234 mg once/month
Paliperidone palmitate	175, 263, 350, 525 mg prefilled syringes	410 mg once per 3 months
Risperidone microspheres	12.5, 25, 37.5 or 50 mg vials	25 mg every 2 weeks

Unfortunately, the use of LAIs also has several negative aspects. The most important are gradual dose titration and the long time required to obtain steady state concentrations [54]. One should also be aware that LAIs make it more complicated to make sensible dose adjustments because the attainment of steady state plasma concentrations may take more than 2 months after a dose change. Therefore, the beginning of therapy with LAIs has generally been confined only to stabilized patients [55]. Administration of LAIs is also associated with the occurrence of several side effects, including postinjection syndrome with olanzapine [56], neuroleptic malignant syndrome (NMS) [57], pain, skin irritation and swelling at the injection site [37,55]. The summary of the most important benefits and deficiencies of LAI administration has been presented in Table 3. The SGA LAIs are better tolerated and present fewer adverse neurological side effects than older FGAs [50].

Another crucial factor is the dosing adjustment of LAI antipsychotics before starting co-administration of other drugs. For instance, paliperidone palmitate and risperidone are contraindicated with simultaneous co-administration of strong CYP3A4 and/or P-gp inducers [32]. On the other hand, the dose of risperidone microspheres should be reevaluated during concomitant fluoxetine or paroxetine therapy initiation. A decrease in the dose of risperidone microspheres has been advised 2–4 weeks before the planned initiation of fluoxetine or paroxetine [59]. In turn, concomitant administration of CYP2D6 substrates or inhibitors may result in increased fluphenazine concentrations [60]. By contrast, co-administration of haloperidol with CYP3A4 inducers, such as rifampin and carbamazepine, may require an increase in the antipsychotic drug dose [61].

One of the most important issues related to LAIs is their effectiveness compared to oral antipsychotics. It was the subject of a few meta-analyses and reviews, which reported conflicting and ambiguous results. For instance, Leucht and co-authors [62] compared data from 1700 subjects and reported that the administration of depot neuroleptics is associated with 30% lower risk of relapse than oral antipsychotic medications. Another meta-analysis, including 21 studies and over 5000 patients, did not report any significant difference with regard to a relapse of schizophrenia in patients receiving LAIs or oral antipsychotics. Generally, the authors did not find an overall advantage for LAIs over oral medicines [63]. In turn, the PROACTIVE (Preventing Relapse Oral Antipsychotics Compared to Injectables Evaluating Efficacy) multicentred, randomised trial on 305 patients treated with LAI risperidone or SGAs did not show a significant difference in the rates of relapse and hospitalisation over 30 months. However, the authors found that patients in the LAI group had greater improvements in some types of psychiatric symptoms [64].

The reasons for the discrepancies between the results of different meta-analyses might stem from several factors, such as changes over the years in the definition of relapse, dissimilarities between older and newer neuroleptics, patient selection, and recent current trends towards the use of lower doses of neuroleptics [34]. 

Studies also compare the effectiveness of FGA LAIs to the SGA depots. Rubio et al. [65] examined 115 subjects with schizophrenia and substance abuse and reported that risperidone LAI-treated patients had substantially fewer positive urine drug tests and lower ratings on a psychotic symptom scale compared with individuals treated with zuclopenthixol LAI. On the contrary, Lammers et al. [66] did not report any important difference between LAI risperidone and FGA LAI treatment in schizophrenia patients. The authors did not report any differences in relation to therapy cessation or hospitalisation, but found a greater incidence of extrapyramidal symptoms in subjects using FGA LAIs [66]. The basic pharmacokinetic characteristic of LAI antipsychotics is presented in Table 4. 

## 4. Formulation Aspects of LAI Antipsychotics

### 4.1. Formulation Technologies of the Marketed LAI Antipsychotics

Generally, the marketed LAI antipsychotic formulations are prepared using one of the three following technologies: (A) polymer-based systems including microencapsulating systems and in situ forming (ISF) polymer implants, (B) oil-based formulations, and (C) drug (nano and micro) suspensions [76]. Oil depot technology has been successfully applied for the first generation of LAI antipsychotics. An oily solution containing an ester form of the respective drug is one of the oldest and most uncomplicated methods for long-acting drug delivery systems (DDS). The product is usually injected undiluted via the intramuscular route to form a sustained-release depot, slowly dissolved into the bloodstream [77]. Esterification of the biologically active molecule to a long-chain fatty acid increases drug’s lipophilicity and solubility in oils, thus allowing for the straightforward manufacturing process and terminal sterilization of the final product [78,79]. The length of the chosen fatty acid influences observed lipophilicity and, consequently, stability of the depot since dissolution of the active substances is preceded by gradual hydrolysis of the ester bond [7,80]. Hence, selection of the fatty acid allows for rational design of sustained-release depots with tailored delivery characteristics. Application of vegetable oils as a vehicle enhances the long-term stability of the formulation because fatty acid esters are essentially not susceptible to degradation processes in oil-based drugs’ storage conditions. Commonly used oils in first-generation LAIs are sesame oil (haloperidol decanoate, fluphenazine decanoate) or fractionated coconut oil (flupenthixol decanoate and zuclopenthixol decanoate) (Table 4) [50]. The products additionally contain a suitable antimicrobial preservative, usually benzyl alcohol. The composition of an oily vehicle determines both density and viscosity of the formulation [79]. The optimal oil vehicle should have relatively low viscosity, which improves the product’s manufacturability and injectability and reduces pain after the injection. In turn, higher viscosity of the composition may impede the sterile filtration process during preparation [81]. Despite the fact that the first generation of LAI antipsychotics based on oil depot technology has been largely superseded by the second generation, fluphenazine decanoate and haloperidol decanoate are still available and commonly prescribed. 

Conversely to the oil-based first-generation neuroleptics, a variety of drug delivery approaches have been utilized for development of the second-generation formulations. These products are administered as suspensions; however, depending on the API’s hydrolytic stability, they are supplied as a ready-to-use suspension (aripiprazole lauroxil, paliperidone palmitate, risperidone microspheres) or as a lyophilized powder and solvent for reconstitution (aripiprazole monohydrate, olanzapine pamoate, risperidone ISD polymer implants). Second-generation neuroleptics can be formulated as flocculated suspensions in order to maintain their physical stability throughout their shelf life or facilitate rapid resuspension. In such suspensions, primary particles agglomerate to form secondary particles, which form loose, porous deposits in the container. Prior to administration, the API particles are resuspended with concomitant disruption of the agglomerates. Creation of an environment enabling flocculation of the API particles is achieved by means of a stabilizing system composed of a surfactant acting as a wetting agent and a polymeric viscosity modifier acting through ionic and/or steric repulsion [82,83,84]. The ionic strength and pH of the solution further modify the properties of this system [85]. It has been found recently that dissolution kinetics and bioavailability of APIs in flocculated suspensions may exhibit dependence on the shear forces applied during administration [86].

In turn, long-acting formulations containing aripiprazole monohydrate are distributed in powder form with an accompanying solvent for reconstitution. Two presentations are available, i.e., glass vials and dual chamber syringes. At the moment of application for Marketing Authorization, the position of the manufacturer was that a flocculated suspension allowed meeting the desired target product’s profile; however, it also caused higher irritation upon administration compared to the deflocculated version. Finally, the deflocculated formulation was promoted. Since a deflocculated suspension generally tends to sediment and irreversibly aggregate, the final product has to be stored as powder [87]. In April 2023 a new ready-to-use form of long-acting suspension containing aripiprazole monohydrate was approved by the FDA [88] (vide infra). Presumably this new presentation is formulated as a flocculated suspension with improved tolerability profile; however, no further details are available. 

Regardless of the chosen suspension type, one of the most common techniques for particle size reduction in crystal suspensions is media milling, specifically bead milling [89]. Overall, the process of preparation of LAI products is much more complicated than traditional formulation and requires several steps which affect the quality of products. In the case of injectable suspensions, these steps are as follows: high shear or pressure homogenization and wet bead milling, while in the case of polymeric microspheres important key manufacturing processes include emulsification, mixing and drying. Another critical factor, common to all particle-based formulations and allowing the achievement of desired drug release properties, is target particle size [76].

This form of pharmaceuticals presents additional quality requirements compared to immediate-release injectable drugs. These characteristics can be divided into three categories: (1) related to patient’s compliance, (2) related to efficacy, and (3) related to safety. Drug loading, injectability, syringeability, or local tolerability can be listed in the first category. Among efficacy-related characteristics, the drug release profile can be listed together with stability or viscosity. Sterility and bioburden are rated as safety issues [76]. Moreover, the in vivo product performance of LAI neuroleptics can be affected by a number of factors, including the attributes of the APIs and the drug product impacting drug release characteristics, as well as numerous physiological factors. Thus, to design and produce an LAI antipsychotic with the appropriate PK profile, it is crucial to develop a set of in vitro, in vivo and in silico tests that would allow for knowledge of the mechanisms of factors influencing LAI product performance. A thorough review of pharmaceutical in vitro analysis together with in vivo and in silico approaches of LAI neuroleptics is presented in Section 5.

Considering a variety of dosage forms developed for LAI antipsychotics, the technological aspects of the selected products along with the details concerning their manufacturing process are presented below.

### 4.2. Formulation Aspects of the Selected Marketed LAI Products

#### 4.2.1. Paliperidone Palmitate LAI

Paliperidone palmitate LAI is marketed by Jansen Pharmaceuticals as a ready-to-use suspension in prefilled cyclic olefin copolymer syringes. Three formulations are available, differing in the required injection intervals: 1 month, 3 months and 6 months. The desired release profile is determined by particle size distribution and paliperidone palmitate dose [90].

The final product is a suspension consisting of the drug paliperidone palmitate and excipients: polysorbate 20, polyethylene glycol 4000, citric acid monohydrate, sodium dihydrogen phosphate monohydrate, disodium hydrogen phosphate (only the 1-month formulation), sodium hydroxide and water for injection. Paliperidone palmitate is present in the suspension as micron- or sub-micron-sized particles. Polysorbate 20 is at the concentration of 10–12 mg/mL, which allows for effective wetting of the paliperidone palmitate particles [91]. Polyethylene glycol stabilizes the suspension by modification of its viscosity. 

The product is manufactured in a process involving: (a) preparation of aqueous vehicle solution, (b) sterile filtration, (c) sterile API addition and homogenization, (d) wet milling, (e) dilution with WFI, and (f) filling and stoppering into presterilized prefillable syringes [90]. Variations of this process are proposed and involve differentiation of the milling medium composition from the final aqueous vehicle so that the milling process is performed for APIs suspended in an aqueous solution of only the wetting agent (polysorbate 20). The remaining excipients are added after milling and another additional step, i.e., aseptic filtration through 40 µm filter [92,93]. Based on the initial particle size distribution of the API, a cascade milling process can be introduced using milling beads of different diameter at consecutive stages [92].

#### 4.2.2. Risperidone LAI Formulations

Risperidone LAI (Risperdal Consta^®^) was the first of the second generation of LAI agents, introduced in 2003 by Jansen Pharmaceuticals [94]. The product applied microsphere technology to incorporate the drug molecule in a biodegradable polymer. Risperidone LAI is available as a single-use dose pack, comprising a vial containing the risperidone microspheres, a pre-filled syringe with a diluent, a vial adapter, and two needles for intramuscular injection (depending on the site of injection) [95]. After mixing the powder containing risperidone microspheres with the aqueous diluent, the drug is administered to the deltoid or gluteal muscle through one needle. The diluent contains sodium carboxymethylcellulose 40 mPa·s, anhydrous citric acid, sodium phosphate-dibasic dihydrate, polysorbate 20, sodium chloride, sodium hydroxide, and water for injection. A simple water-based solvent extraction technique is used to encapsulate the risperidone in a copolymer of poly(glycolic acid) and poly-D,L-lactic acid (PLGA, poly(D,L-lactide-co-glycolide)) [96,97]. 

PLGA, a synthetic aliphatic polyester, is sensitive to hydrolytic degradation into short-chain alcohols and acids. The drug release from PLGA-based products can be tailored by changing the molar ratios of lactic acid to glycolic acid, or by the molecular weight of PLGA. The higher molecular weight contributes to reduced polymer degradation, and subsequently slower drug release [76].

The process of Risperdal product manufacturing involves a few steps: (1) preparation of an aqueous phase containing polyvinyl alcohol (PVA) and an organic phase with PLGA and risperidone; (2) emulsification of the organic polymer solution in an aqueous continuous phase; (3) droplet formation by means of static mixing, and (4) quenching and isolation of the microspheres via sieving. Drug release and drug encapsulation efficiency are governed by the size and size distribution of the resulting microspheres, and these physicochemical parameters essentially depend on the droplet formation step [96]. 

Also, recently tremendous efforts have been made toward developing risperidone formulations. In 2018, the FDA approved a new formulation of risperidone (Perseris^®^) for the treatment of schizophrenia in adults [98]. The superiority of this once-monthly, extended-release, subcutaneously-administered sterile depot formulation over the biweekly Risperdal Consta is achieving clinically relevant therapeutic plasma concentrations of risperidone on the first day of dosing, with no need for a loading dose or supplemental oral dosing [99]. Perseris^®^ is available in two different strengths, which deliver 90 mg or 120 mg of risperidone over one month. It is an ISF polymer implant using the Atrigel^®^ drug delivery system with poly (DL-lactide-co-glycolide) polymer and N-methyl-2-pyrrolidone (NMP) as a solvent [98]. The product is supplied as a drug–device combination product in two separate pre-filled syringes. The liquid-containing syringe is filled with the colourless to yellow Atrigel^®^ solution, whilst the powder-filled syringe contains the lyophilized drug powder. The liquid-filled syringe contains 228 mg PLGA (L/G 80:20) dissolved in 282 mg NMP for the 90 mg/kit strength and 304 mg PLGA (L/G 80:20) dissolved in 376 mg NMP for the 120 mg/kit strength. Prior to administration, both syringes are connected through luer lock fittings and the content is passed back and forth to obtain the suspension. Afterwards, the combined mixture is transferred into the liquid syringe and the two syringes are decoupled, followed by the attachment of a safety needle for injection [100]. The designed two-syringe system not only ensures efficient mixing of the suspension but also allows the product to be sterilized by gamma irradiation and increases its stability. 

Apart from Risperdal Consta and Perseris^®^, in May 2023 the FDA approved another risperidone formulation (Uzedy, Teva and MedinCell), an extended-release injectable suspension for the treatment of schizophrenia in adults. The injection applies technology to control the steady release of risperidone, and therapeutic blood concentrations are reached within 6 to 24 h of a single dose. Uzedy is a sterile, white to off-white opaque viscous suspension available in the following strengths: 50 mg/0.14 mL, 75 mg/0.21 mL, 100 mg/0.28 mL, 125 mg/0.35 mL, 150 mg/0.42 mL, 200 mg/0.56 mL, and 250 mg/0.7 mL. Each strength is provided as a kit, which includes one single-dose prefilled syringe and one 21 gauge, 5/8-inch needle. The product offers flexible 1- and 2-month dosing intervals [101]. This pharmaceutic utilizes a copolymer technology (a biodegradable copolymer having the formula poly(lactic acid)_v_-poly(ethylene glycol)_w_-poly(lactic acid)_x_, and a diblock copolymer having the formula methoxy-poly(ethylene glycol)_y_-poly(lactic acid)_z_), that controls the rate and duration of drug release [102,103]. In comparison to the original risperidone formulation (Risperdal Consta), the UZEDY product contains inactive ingredients such as dimethyl sulfoxide (45% *w*/*w*) and the above-mentioned polymers methoxy-poly(ethylene glycol)-co-poly(D,L-lactide) (15% *w*/*w*), and poly(D,L-lactide)-co-poly(ethylene glycol)-co-poly(D,L-lactide) (10% *w*/*w*) [101]. 

Moreover, Rykindo is a new LAI formulation of risperidone, approved by the FDA in 2023. This product is marketed as a lyophilized powder and applies microsphere technology to deliver long-acting and extended-release risperidone [104]. The powder is composed of risperidone, mannitol, polylactide-co-glycolide (PLGA) 5050 and polylactide-co-glycolide (PLGA) [104]. The solvent for reconstitution is composed of citric acid monohydrate, dibasic sodium phosphate anhydrous, polysorbate 80, sodium carboxymethyl cellulose, sodium chloride, sodium hydroxide, and water. Notably, Perseris, Uzedy and Rykindo may serve as excellent examples of the crucial role that biodegradable polymers, especially PLGA derivatives with tailored drug delivery properties, play in the LAI formulations today [105,106]. 

Recently, Laboratorios Farmacéuticos ROVI, S.A., has also designed a new injectable formulation of risperidone using the in situ microparticles (ISM) technology which is based on an in situ forming solid polymeric matrix system containing risperidone. The suspension obtained after reconstitution results in an early, rapid, and sustained release of the drug by 2 h after its administration and lasts for 1 month [107]. The results of clinical studies showed that risperidone formulated in ISM technology provides rapid and progressive reduction in symptoms in subjects with acutely exacerbated schizophrenia without the necessity of additional oral drug supplementation [107].

#### 4.2.3. Aripiprazole Monohydrate LAI

Aripiprazole monohydrate long-acting injection is marketed by Otsuka Pharmaceutical as a sterile, single-dose, lyophilized powder for prolonged-release injectable suspension and solvent for reconstitution (water for injections). The powder is composed of the active ingredient, aripiprazole monohydrate particles and excipients: sodium carboxymethyl cellulose (suspending agent), mannitol (bulking agent), sodium dihydrogen phosphate monohydrate (buffering agent), and sodium hydroxide (pH adjustment promoter). The solvent for reconstitution is composed of water for injections with no other excipients [87].

The product in vials is manufactured in an aseptic process involving: (a) preparation of the aqueous vehicle solution, (b) sterile filtration, (c) sterile API addition, (d) wet milling, (e) filtration, (f) filling to vials and half-stoppering, (g) lyophilization in vials, (h) full-stoppering and capping [87,108]. Due to the low volume offered by double-chamber syringes in order to ensure effective resuspendability, the manufacturing process reportedly requires the lyophilization process to be performed as freeze spray drying [109]. The wet milling step can be replaced by high pressure homogenization [110]. In the bead milling step, instead of conventionally used zirconium beads, polymeric beads were shown to be effective in a laboratory setup [111].

In August 2023, the FDA approved an NDA for ABILIFY ASIMTUFII^®^, aripiprazole monohydrate LAI in the form of a ready-to-use aqueous suspension containing 960 mg of aripiprazole monohydrate for the treatment of schizophrenia in adults or for maintenance of monotherapy treatment of bipolar I disorder in adults [112]. The excipients include carboxymethylcellulose sodium, polyethylene glycol 400, povidone, sodium chloride, sodium phosphate monobasic monohydrate, sodium hydroxide (to adjust pH) and water for injection [113]. Clinical trials demonstrated that the safety, efficacy, and pharmacokinetic profile of the 2-months formulation are similar to those of aripiprazole once-monthly 400 mg doses [114]. In turn, in May 2023 Otsuka Pharmaceutical withdrew its application for a marketing authorization of Asimtufi in the European Medicines Agency. Based on the review of the data, the EMA noted that in the main study the company should have compared Asimtufii with the reference medicine (Abilify Maintena) available in the EU. According to the EMA opinion, without these clinical results, the study presented by the company did not provide enough evidence to support the application. Conversely, Otsuka explains the withdrawal of its application by a change in the company’s strategy [115].

#### 4.2.4. Aripiprazole Lauroxil LAI

Aripiprazole lauroxil was introduced to the pharmaceutical market in 2015 by Ireland-based Alkermes. Aripiprazole lauroxil is an injectable antipsychotic with one-month and two-month formulations, used to manage schizophrenia [116]. The excipients used in the intramuscular injections in a form of suspension are as follows: polysorbate 20, sodium chloride, sodium phosphate dibasic (anhydrous), sodium phosphate monobasic (dihydrate), sorbitan monolaurate, and water for injections. Extended-release aripiprazole lauroxil is available in 441-, 662-, 882- and 1064-mg strengths in prefilled syringes (Aristada), and in the form of 675-mg strength prefilled syringes (Aristada Initio). Both of these preparations are ready-to-use aqueous suspensions of crystalline particles. The volume of injection depends on the dose. Ready-to-use suspensions take advantage of the low solubility of the drug to prolong shelf life. When combined with a solution, solid particles are uniformly distributed, making the preparation cloudy or blurred [117]. The formulation has been developed in a way that the drug molecules are loosely associated. This results in a shear-thinning property contributing to lower viscosity during rapid injection, allowing the suspension to flow easily through the needle [117].

#### 4.2.5. Olanzapine Pamoate

Crystalline drug suspension technology was applied in olanzapine pamoate, a microcrystalline salt of olanzapine and pamoic acid suspended in a long-acting formulation. The drug is marketed by Eli Lily as a powder and solvent for prolonged-release suspension for injection. It is available in three different strengths, which deliver 210 mg, 300 mg, or 405 mg of olanzapine over 2 weeks (for 210 mg and 300 mg) or 4 weeks (300 mg and 405 mg). The powder is composed of the active pharmaceutical ingredient olanzapine pamoate monohydrate salt, with no additional excipients. The solvent for reconstitution consists of sodium carboxymethyl cellulose, mannitol, polysorbate 80, sodium hydroxide, hydrochloric acid, and water for injections [118]. The necessary volume of the solvent for reconstitution is determined by the dose so that after reconstitution 1 mL of the suspension of each presentation contains 150 mg olanzapine. Properly reconstituted olanzapine pamoate is a yellow opaque suspension.

The manufacturing process of the powder involves dry-milling of the olanzapine salt to achieve the target particle size distribution, filling into vials, sealing and terminal sterilization via electron beam radiation. The solvent for reconstitution is manufactured by dissolution of the constituents, pH adjustment, sterile filtration, filling to vials and terminal sterilization [118].

## 5. Pharmaceutical Analysis of LAI Antipsychotics

### 5.1. Drug Specification through Analytical Tests

All drugs are subjected to thorough analytical control. Depending on their properties, formulation type and risks associated with their use, various sets of tests are arranged in the form of product specification. According to ICH guidelines, a specification is described as a set of tests, references to analytical techniques, and acceptance requirements, including numerical limits, ranges, or other criteria for the described tests [119]. It specifies the list of requirements that a pharmaceutical must meet in order to be regarded as suitable for its appropriate purpose. Additionally, the specifications are crucial quality criteria established and justified by the manufacturer and authorized as conditions of approval by the regulatory agencies. Defining critical attributes and criteria to control them correctly are the two most important components of specification setting. The drug product specifications should focus on those qualities that are critical to assure safety and efficacy rather than establishing exhaustive characterization [120]. The exemplary specification of the product for SR injection is presented in Table 5. 

#### 5.1.1. Appearance

Each drug, before more complex tests, undergoes a simple visual assessment and is compared to the model description. As part of it, the analyst can assess the time of reconstitution (for drugs in the form of a lyophilizate), syringeability (aspiration into syringe), injectability (ease of injection) and particulate contamination visible with the naked eye [120].

#### 5.1.2. Identification

Identification of the API as well as some of excipients can be performed using different methods. If possible, it is practical to use a method that determines the assay or purity of an active substance, such as the HPLC method. A second method can be incorporated to increase the level of certainty [120]. 

#### 5.1.3. Water Content

In the case of formulations in the form of a lyophilizate, the correct level of water at release proves the accuracy of the lyophilization process. During stability tests, the control of the water content allows confirmation that changes do not occur during storage. This is vital information about an appropriate drug closure system [120].

#### 5.1.4. Particle Size Distribution

Particle size distribution influences release rate. In a suspension containing particles of ca. 2 μm and secondary flocculates of tens of microns, deflocculated particles dissolve up to six times faster than the flocculated particles [86]. Controlling this parameter is crucial during development of drug product formulation. Later it serves to confirm the reproducibility of the manufacturing process.

#### 5.1.5. In Vitro Release

The need for release testing emerged in the 1950s with the discovery of the relationship between bioavailability and dissolution. In 1970 Apparatus 1 (Basket Apparatus) was adopted, followed by Apparatus 2 (Paddle Apparatus) in 1978 and Apparatus 4 (Flow-Through Cell) for extended-release products in 1995 [121]. Despite a long history of LAIs, the field of their dissolution is still in its infancy with the lack of a standard method for the assessment of the LAI release profile. The lack of generalized world-wide accepted in vitro release methods and process optimization also impedes the regulatory approval process.

With the increasing diversity of new drug formulations, release methods have become more and more product specific. Pharmacopoeial apparatuses are still in use, but alternative methods, such as dialysis methods, have been developed and are accepted as long as their adequacy can be demonstrated. The USP 4 flow-through cell method is often an appropriate choice for in vitro release method of long-acting parenteral (LAP) formulations of hardly water-soluble drugs. Apparatus 4’s advantage is the greater similarity of hydrodynamics to in vivo and the ease of providing sink conditions [122]. Apart from in vitro dissolution studies, there are also devices allowing for simulations of drug behaviour after subcutaneous injection. One such device is the SubCutaneous Injection Site Simulator system (SCISSOR), where the drug is injected into a cartridge representing the extracellular matrix (ECM) to simulate the in vivo conditions [123]. The ECM is built-in in a dialysis chamber which can be filled with various solutions and concentrations of constituents and which is immersed in another chamber filled with bicarbonate-based physiological buffer to simulate drug migration from the injection site into the blood capillaries [123]. The SCISSOR system provides information on the possible interactions occurring between the LAI and the subcutaneous environment in a physiologically relevant way, and aims to examine the potential fate of a biopharmaceutical after its SC injection. Therefore, the system provides an alternative to animal testing. Using appropriate media and constituents, inflammatory tissue reactions or injection site reactions after biopharmaceutical injection might be simulated [123]. 

Many parameters, including particle size, morphology, the crystallinity of the API, residual solvent in the API, and excipient source, might influence the release of substances from LAI suspensions [124]. Description of the drug dissolution profile of LAI products is required to assess proper quality and the appropriate in vivo effects [125]. A few years ago, USP published a draft chapter entitled ‘In vitro release test methods for parenteral drug preparations’ to supply a validated knowledge on in vitro release methods to determine the performance of parenteral drug products [76].

**Figure 3:**
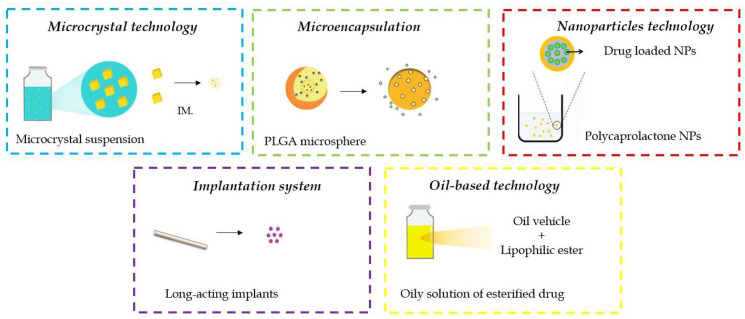
Examples of drug release modifications in parenteral formulations. IM—intramuscular; NPs—nanoparticles.

Release rate studies are utilized to assess the quality of orally applied pharmaceuticals. Therefore, there is a necessity to develop the appropriate method to test in vitro release of LAI formulations. However, despite the increasing use of such preparations, also in psychiatry, there is no officially approved method for long-term release parenteral formulations. The development of new methods for in vitro release of LAIs is carried out to predict the drug availability in the early steps of product development, to establish formulation factors and manufacturing methods of dosage form, and to meet regulatory requirements [125]. Furthermore, in vitro dissolution studies enable the estimation of in vivo drug concentrations and behaviour. In vitro test conditions for paliperidone, aripiprazole and olanzapine can be found on the FDA website (http://www.accessdata.fda.gov/scripts/cder/dissolution/ (accessed on 25 September 2023)). Several examples of drug release methods in various pharmaceutical preparations of LAI antipsychotics are given below, and depicted in Figure 3.

##### Aripiprazole Sustained-Release Microcrystals for Intramuscular Injection

To measure drug release in vitro, a drug transdermal diffusion tester was employed. The diffusion cell’s receiving capacity was 7 mL. The samples were stirred at 300 rpm at 37 °C. The dissolving medium was 0.25% sodium dodecyl sulfate, and the samples were withdrawn at appropriate intervals (30 min, 1 h, 4 h, 6 h, 8 h, 12 h, 24 h, 48 h, and 72 h). The quantitative analysis was conducted using the HPLC method [111]. In turn, in vitro dissolution conditions for aripiprazole in a form of intramuscular suspension are as follows: paddle apparatus (USP II), centrifugation speed 50 rpm, 0.25% Sodium Dodecyl Sulfate (SDS) Solution (900 mL) as medium, and recommended sampling times 10, 15, 30, 60, 120, 180, 240, 300, 360, 420, and 480 min [126].

##### Poly(D,L-lactide-co-glycolide) (PLGA)-Based Microsphere Formulation

Microsphere formulations need specific methods for their characterization, for instance: surface morphology, shape, size, bulk density, encapsulation efficiency, Fourier transform infrared spectrometry and in vitro release. The release of medicine from PLGA microspheres is affected by polymer type and molecular weight, process variables, drug characteristics, and microcapsule particle size. The low molecular weight of PLGA polymer promotes drug diffusion, polymer erosion, and water permeability of PLGA microspheres. Reduced microsphere size enhances the surface/volume ratio, which increases drug diffusion from microspheres. As the polymer ratio in the organic phase increases, the thickness of the polymer wall and the size of the microsphere rise, resulting in a slower rate of dissolution [127]. Olanzapine was successfully incorporated into PLGA microspheres, and in vitro drug release was established using a shaker water bath. The samples were obtained at certain time points, and the drug concentration was determined using the reverse phase HPLC method. In vitro release of the drug was substantially influenced by formulation parameters, including polymer type and molecular weight, process variables, properties of the drug and particle size of the microcapsule. For instance, changing the ratio of lactic to glycolic acid (50:50, 65:35, 75:25, 85:15) in PLGA resulted in decreased drug release. Furthermore, reducing the size of microspheres elevated the surface/volume ratio, which contributed to the increased diffusion of olanzapine [127]. In turn, the FDA’s proposed dissolution test conditions for olanzapine pamoate for intramuscular suspension differ. The FDA advises using the USP IV apparatus with flow at 3 mL per minute, and 1% SLS in pH 6.8 phosphate buffer as medium. Recommended sampling times range from 10 to 720 min [128].

PLGA microspheres were also used to incorporate another neuroleptic, norquetiapine. The in vitro dissolution test of norquetiapine PLGA microspheres was conducted by suspending 10 mg of them in 10 mL of phosphate-buffered saline (PBS, pH = 7.4). The test was carried out for 20 days at 37 °C ± 0.5 °C in a rotary shaking incubator at 50 rpm. The supernatant was separated for a validated RP-HPLC analysis at each preset sample point, and fresh medium of equal volume was added [129]. The drug was released over 20 days, with a typical biphasic release pattern consisting of initial burst release and a more controlled secondary phase [129].

In order to design a new 3-month releasing formulation, risperidone microspheres were evaluated with a series of tests. Particle size was measured by an optical microscope. Thermal behaviour was investigated with differential scanning calorimetry. A scanning electron microscope was applied to determine surface morphology and shape. Efficiency of drug entrapment was calculated by comparing the theoretical and practical drug content which was determined by UV spectroscopy. Drug release was monitored for a period of 90 days. In microtubes, 10 mg of microspheres were suspended in 1 mL of 7.4 pH buffer solution. These microtubes were shaken in a horizontal water bath at 37 °C. At the end of specific time periods, these microtubes were put in a centrifuge set at 5000 rpm for 10 min then 1 mL of the supernatant solution was withdrawn, filtered, and diluted to 5 mL with buffer. The reader should be aware of the potential effects of centrifugation conditions on the microsphere integrity and drug release. The higher the centrifugation speed, the greater the probability of changing the integrity of the microspheres and the greater the drug release. The increased centrifugation speed is associated with an increase in the centrifugal force acting on the fractions of microspheres with biologically active substances enclosed in these fractions, even leading to the destruction of microspheres and changes in release profiles. The design of accelerated methods dedicated to routine quality control should be correlated with multi-day tests, taking into account the specificity of the drug form and the expected release profile. This becomes particularly important in studies of sustained-release formulations [130]. The withdrawn volume was refilled with an equivalent amount of new buffer solution, the microsphere suspension was resuspended, and the release study was resumed. The quantity of risperidone released was measured using a UV spectrophotometer at 278 nm and distilled water as the blank. By plotting % cumulative drug release vs time, the dissolution characteristics of the formulations were compared. The in vitro behaviour of risperidone microspheres exhibited prolonged and sustained release of the drug [131]. The type of polymer affected the drug release with PLGA-based microspheres showing slower release when compared to that of polycaprolactone-based microspheres [131]. These examples show that using real-time release methods that take many days is possible during product development, but accelerated methods are necessary for routine quality control. 

##### Paliperidone (PP) Loaded Polycaprolactone (PCL) Nanoparticles

The FDA proposes the application of USP II Paddle apparatus for the dissolution studies of paliperidone palmitate in intramuscular suspension (both 1 and 3-mont injections). The acceptance medium is 0.489% (*w*/*v*) Polysorbate 20 in 0.001 N HCl at 25.0 °C for both formulations. The method differs only in the time intervals. In turn, methods described in the literature present different protocols and conditions [132]. For instance, in vitro release studies of paliperidone were conducted using a dialysis method using a screw-capped Spectra/Por^®^ dialyzing tube (Float-A-Lyzer^®^G2). For 30 min, the tubes were soaked in purified water. A dose of 10 mg PP was placed inside the dialyzing tube, which was immersed in a tightly closed glass beaker containing 400 mL of phosphate-buffered saline (PBS) of pH-7.4 on a magnetic stirrer (37 ± 2 °C, 100 rpm). At adequate time intervals, 2 mL aliquot was withdrawn from the release medium and diluted with acetone/ethyl acetate mixture (1:1, *v*/*v*) and the percentage of PP released was assessed spectrophotometrically (323 nm). After 28 days, the total amount of PP released from various formulations was statistically compared [133]. Prepared formulations were characterized by biphasic drug release pattern with the highest release through the first 4 days followed by sustained release afterward, associated with the slow diffusion of drug particles across the polymeric matrix. Formulations coated with chitosan released the drug in a much slower manner in both stages compared with the analogous uncoated formulations, which was due to the electrostatic interaction of positively charged chitosan with negatively charged stabilizers in slightly acidic media producing a gel [133].

##### Risperidone Controlled-Release Microspheres Based on Poly(Lactic acid)-Poly(Propylene adipate)

Another example of dissolution studies of LAI neuroleptics was reported by Nanaki et al. [134] who evaluated the preparation of risperidone controlled-release microspheres based on new PLA/PPAd polymer blends. For the in vitro release studies, the basket method was used (United States Pharmacopeia, North Bethesda, MD, USA, USP I method). Microspheres were introduced into dissolution baskets using appropriate dialysis tubing cellulose membranes. The release study was carried out at 37 ± 1°C with a rotation speed of 50 rpm. The dissolution medium was 500 mL of a phosphate buffered saline (PBS) solution with pH = 7.4. An aliquot of 2 mL was taken from the release media at appropriate time intervals and analysed for risperidone content by HPLC [134].

Prepared microsphere products exhibited much higher dissolution release rates in comparison with pure risperidone. This phenomenon was due to the amorphous or molecular-level dispersion of risperidone inside the polymer matrix. The drug was released from microspheres in a two-step manner, with an initial burst release phase, lasting up to six hours, and a controlled-release phase till the end of the dissolution process [135]. As the major limitation of original intramuscular suspension of risperidone (Risperdal Consta) is approximately 3 weeks’ release lag time for achieving the therapeutic drug levels, several new approaches, including PLGA-based microspheres [135,136], microspheres co-encapsulated with magnesium hydroxide or arginine base [137], and PLGA-based in situ depot systems [138], have been developed and reported with various results.

##### Implantation Systems

Yan et al. [139] evaluated the role of different pore formers, polymer ratios, porogen levels, and oil–water ratios on risperidone release from implantation systems. The authors used cumulative release, drug loading, and entrapment efficiency as assessment indicators. The researchers looked at crystalline alterations, residual solvents, solubility, and stability following sterilization, in-vivo polymer degradation, pharmacokinetics, and tissue inflammation. The in vitro release test was performed with a constant temperature shaker (37 ± 0.5 °C) and rotation speed of 50 rpm. The medium contained 80 mL of PBS (pH 7.4). Samples were collected on the first day and then every three days from the third day, with the medium being replaced each time. The implantation system released risperidone with zero-order kinetics in vitro. The authors reported that the average daily release rate of the drug was stable (0.6–1.8%), and the primary average daily release rate was 1.0%. At the beginning of the dissolution studies, the PLA was less affected by the medium, and the average daily dissolution rate was maintained. However, after 20 days, the PLA on the surface of the implant was eroded, and the average daily release of the drug was elevated [139]. 

## 6. Predictive In Vivo Performance—In Vitro–In Vivo Correlation (IVIVC)

The definition of an in vitro–in vivo correlation (IVIVC) by the U.S Food and Drug Administration (FDA) states that it is a predictive mathematical model describing the dependence between the in vitro property of a drug or a dosage form (rate or extend of the drug release/dissolution) and adequate in vivo response meaning the plasma drug concentration [140].

For the development of an IVIVC, in vivo drug properties can be predicted from its in vitro release profile. Therefore, the developed and applied IVIVC can be treated as a replacement of bioequivalence studies, and reduce pharmaceutical regulations [140]. IVIVC is also sometimes replaced in the scientific literature by the term in vitro—in vivo relationship (IVIVR), but generally it relates to the same issues of drug dissolution and pharmacokinetic parameters [141]. 

Performing in vitro dissolution tests with in vivo relevance has several advantages, including cost reduction of conducting in vivo studies and acceleration of pharmaceutical development [125]. Thus, investigation of the IVIVC has recently become an important part of LAI formulation development. Unfortunately, at the moment of manuscript preparation, there is no regulatory IVIVC guidance available for LAI pharmaceuticals. Actually, the principles of IVIVC guidance regarding oral extended-release drug products have been employed for parenteral LAIs. Numerous in vitro dissolution methods have been designed to characterize drug release from LAIs; despite this, there are very few IVIVC neuroleptics cases reported in the literature.

Rawat et al. [142] reported the relationship between in vitro and in vivo release of available Risperdal^®^ Consta^®^ microspheres. For in vitro release testing, a modified USP apparatus 4 (Sotax CE7 smart with CY 7 piston pump, Sotax, Horsham, PA, USA) was used. The authors used flow through cells packed with 9 gm of glass beads. Filling one-third of the cells with glass beads prepared the flow through cells. Ten milligrams of microspheres were weighed and divided into three groups. The first part was placed over the glass beads in the cells, followed by one small scoop of glass beads. The same protocol was applied the rest of samples. The cells were completely filled with the leftover glass beads. At a flow rate of 8 mL/min, 250 mL of 0.05 M PBS pH 7.4 with 0.1% sodium azide was circulated through the cells. For the real-time studies, the temperature was kept at 37 °C, while the accelerated in vitro release tests were performed at an elevated temperature. At appropriate time intervals, one millilitre samples were collected and replaced with fresh medium. The probes were analysed using the HPLC method [142]. 

The authors reported a biphasic in vitro release profile of risperidone, after an initial increased release at 37 °C. The release duration reduced from ca. 40 days (at 37 °C) to 7 days under accelerated conditions (45 °C) and a good correlation (R^2^ = 0.9929) was observed between the two profiles after time scaling. The in vivo plasma profile of risperidone obtained in clinical studies was deconvoluted for comparison with the in vitro release profiles. Both in vitro release and in vivo absorption profiles exhibited a lag phase of similar duration (∼24 days) followed by a steady increase in drug release/absorbtion after day 24. The in vitro dissolution of the drug in the form of microspheres was terminated in ca. 40 days whereas the plateau was reached in 56 days in the absorption studies. The authors reported that in vivo absorption was faster than the in vitro release up to 30 days but afterwards it slowed down. Importantly, the researchers estimated a correlation coefficient for the fractions dissolved in vitro and absorbed in vivo, which was 0.93 [142]. 

Another example of successful IVIVC was published by D’Souza et al. who reported an almost linear correlation (r^2^ = 0.96) between in vitro dissolution and in vivo release in a rat model of olanzapine-loaded PLGA microparticles [143]. Promising results were also obtained by Park et al. who reported a favourable IVIVC (r^2^ > 0.98) of PLGA microparticles containing norquetiapine [129].

Another way of predicting the pharmacokinetics of LAI neuroleptics is physiologically-based pharmacokinetic (PBPK) modelling, which was developed to assist product design, the selection of optimal dose or the dosing regimen. Importantly, PBPK modelling can also be used to predict potential drug–drug interactions with concomitant medications [76].

## 7. Future Perspective

From the regulatory and commercial perspective, it reasonable that the marketed LAI products are mainly a reformulation or repurposing of the drugs already on the market [144]. The usage of these strategies is beneficial during the registration process since a simplified standard document of common elements (CTD) can be submitted to the FDA, EMA or a national Regulatory Agency. More detailed review of the regulatory aspects can be found elsewhere [145,146]. Reformulation of the “old drug” strategy has also been observed for the LAI antipsychotics [144]. From the regulatory and commercial perspective, it is reasonable that the marketed LAI products are mainly reformulations of the drugs already on the market [144]. This trend is also observed for the LAI antipsychotics. Thus, the recently approved or submitted for approval oral antipsychotic drugs may serve as a platform for the future development of LAI formulations with superior performance. In the past few years, the U.S. Food and Drug Administration (FDA) has approved several new antipsychotic drugs. One of them is cariprazine, approved in 2015, which is a derivative of 2,3-dichlorophenylpiperazine. The drug is indicated for the treatment of schizophrenia and acute manic or mixed episodes associated with bipolar I disorder in the form of a monotherapy. In terms of pharmacodynamics, cariprazine is similar to other SGAs, and acts antagonistically towards 5HT_2A_ receptors [147]. Furthermore, the drug is a partial agonist towards D_2_ and D_3_, and 5HT_1A_ receptors. Importantly, cariprazine possesses a unique property, namely it shows a higher affinity for the D_3_ than the D_2_ receptor. However, the clinical significance of this feature is unknown. Regarding other interactions with receptors, cariprazine also shows moderate histamine antagonism, and low α_1_-antagonism. The drug does not exhibit a substantial affinity towards muscarinic receptors. Cariprazine is metabolized into two pharmacologically active derivatives, namely desmethyl and didesmethyl cariprazine, of which the latter is accountable for long-term efficacy and tolerability [147]. Cariprazine is primarily available in oral tablet form. It is marketed under different brand names depending on the country, such as Vraylar in the United States and Reagila in Europe. The current drug delivery method has only immediate-release oral dosage forms, which is not convenient to all types of patients suffering schizophrenia. However, attempts to design new formulations including a long-acting formulation or extended-release drug delivery system are already under way. For instance, Hui et al. [148] reported novel formulations of cariprazine such as spheres, microspheres or nanoparticles where the active ingredient can be ionically complexed with the biodegradable and biocompatible polymer or the active agent is dispersed in the matrix material.

Another new antipsychotic drug is lumateperone, a derivative of butyrophenone, which was registered with the FDA on 20 December 2019. It received FDA approval for the treatment of schizophrenia in adults. Its exact mechanism of action is not fully studied, but it is believed to involve a combination of antagonism and partial agonism at various receptors in the brain. Lumateperone primarily acts as an antagonist at the 5-HT_2A_ receptor, and has a partial agonist activity at D_2_ receptors. It acts as a postsynaptic D_2_ receptor modulator, which means it can either stimulate or inhibit dopamine signalling depending on the level of dopamine activity in the brain. The drug also has a moderate affinity for α_1_ and H_1_ receptors [149]. Lumateperone, marketed under the brand name Caplyta, is available in oral capsule form. In 2022, a clinical trial of phase I (NCT04709224) was completed, the aim of which was to determine the pharmacokinetics, safety and tolerability of single ascending doses of a subcutaneous LAI of lumateperone. Unfortunately, the results of this study are not available [149,150]. At the time of writing this paper, data on extended-release formulations or LAIs were not found.

Brexpiprazole was approved for the acute and maintenance treatment of schizophrenia in 2015. Brexpiprazole acts as a partial agonist at several key receptors, including dopamine D_2_ and serotonin 5-HT_1A_, and an antagonist at 5-HT_2A_ receptors. As a partial agonist, it can both stimulate and inhibit the activity of these receptors, depending on the level of neurotransmitter activity in the brain. It is important to note that the exact mechanisms of action of brexpiprazole and its effects on different receptor systems are still being investigated, and further research is needed to fully understand its precise mode of action [151]. Brexpiprazole is available in oral tablet form, and similarly to lumateperone there is no information on the marketed sustained-release formulations. In 2021, a clinical study (NCT05119894) of phase I was started to investigate the pharmacokinetics, tolerability, and safety of brexpiprazole LAI administered as a single dose in patients with schizophrenia. However, in 2022 the study was withdrawn due to the changes in the development plan [152]. According to the patent information, the brexpirazole LAI is a ready-to-use form of long-acting suspension containing brexpirazole dihydrate as an active substance, sodium chloride, polyethylene glycol 400, polysorbate 80, and phosphate buffer [153].

More recently, in June 2021, the FDA approved a two-component pharmaceutical containing olanzapine and samidorphan for the treatment of adults with schizophrenia and/or bipolar I disorder. The approval was based on the results of the ENLIGHTEN clinical development program. These studies showed a more statistically significant reduction in weight gain than olanzapine alone in patients with schizophrenia [154]. The new product is available in the form of oral tablets in fixed dosage strengths composed of 10 mg of samidorphan and 5, 10, 15 or 20 mg of olanzapine. According to the manufacturer, the excipients include colloidal silicon dioxide, crospovidone, lactose monohydrate, magnesium stearate, and microcrystalline cellulose [155].

When writing about the latest achievements in the field of antipsychotic drugs, it is impossible to omit the ongoing research in the field of medicinal chemistry. Owing to the complexity of schizophrenia, the current trend in medicinal chemistry is to design a novel chemical entity with a “selective” multireceptor profile which interacts with several specific targets to get better safety and treatment efficacy. For instance, Wu et al. [156] reported synthesis of new arylpiperazine derivatives with quinolinone-like moieties. One of the obtained structures, a compound with benzo[d]isothiazole moiety, was characterized by a promising antipsychotic profile, combining partial agonistic activity for D_2_ receptors, agonistic activity for 5-HT_1A_ receptors, and antagonistic activity for 5-HT_2A_. Importantly, this compound did not exert any significant activity on targets associated with side effects. In vivo studies showed that the compound exerted antipsychotic and antidepressant activity and was characterized by favourable pharmacokinetic [156]. In turn, Huang et al. [157] reported the design and synthesis of a series of benzoxazole–piperidine (piperazine) derivatives exhibiting affinity for D_2_ and 5-HT_1A_, 5-HT_2A_ receptors. One of these compounds was characterized by highly demanding properties, including high affinities for the D_2_, 5-HT_1A_, 5-HT_2A_, 5-HT_7_, and α_2_ receptors, and low affinity for the 5-HT_2C_, H_1_, and α_1_ receptors. These results suggest that the compound could be a promising candidate for further development as an antipsychotic drug [157].

In the world literature, we can also find a new trend regarding the search for compounds acting on metabotropic glutamate (mGlu) receptors. Among them, the mGlu_7_ subtype was reported in the cerebral cortex, hippocampus, amygdala, and basal ganglia, which make these receptors a promising therapeutic target for numerous CNS disorders, such as schizophrenia, depression, or anxiety. Recently, two potent, highly selective mGlu_7_ modulators, VU6019278 and VU6027459, were synthesized and examined [158]. These results highlight the complexity of schizophrenia, and engagement of multiple receptors in the pathogenesis of this disease. It is also important to note that the activity and mechanism of action of newly developed anti-psychotics is not fully understood, and the researchers emphasize their impact on multiple molecular signalling pathways affecting the regulation of growth factors, brain inflammation, and/or immune response [159].

## 8. Conclusions

LAIs neuroleptics, both FGAs and SGAs, remain a potent drug therapy for subjects suffering from persistent psychiatric disease. The significant development of pharmaceutical sciences contributed to the approval of several LAI second-generation antipsychotics on the pharmaceutical market. These are aripiprazole monohydrate, aripiprazole lauroxil and aripiprazole lauroxil loading dose (Initio), three paliperidone palmitate formulations administered every 1, 3, or 6 months, risperidone products (microsphere suspension and subcutaneous injection), and olanzapine. These drugs and their formulations differ in cost, dosing interval, oral overlap requirements upon initiation, dose adjustment requirements for renal/hepatic impairment, and methods of administration [52]. 

One of the primary methods of pharmaceutical analysis is to assess the release profile of the drug from the pharmaceutical formulation. In the case of parenteral drugs, including LAI antipsychotics, no universal method has been described in the pharmacopoeia or other documents regarding the assessment of drug substance release. Herein is presented the analysis of the available literature on the methods of neuroleptics dissolution from the extended-release injectable formulations which confirms the complexity of drug dissolution studies in this case. This, in turn, leads to the problems with obtaining optimal in vivo release profiles. In order to solve this problem, an IVIVC can be performed. However, the need for the adequate regulating body for IVIVC will certainly allow for the thorough investigation of the LAI antipsychotics.

In summary, the development of LAI antipsychotics is more challenging than other drug delivery systems in terms of cost, method of preparation and pharmaceutical analysis. The in vitro drug release profile with burst release and subsequent sustained release, choice of the adequate dose, and IVIVC regulations development are the current limitation factors in the design and development of LAI antipsychotics.

## Figures and Tables

**Table 3:** Summary of benefits and difficulties associated with the administration of LAI antipsychotics.

ADVANTAGES	
Administration once per two or four weeks	
Greater patient compliance	[45,46]
Administration transparency	[55]
Lower probability of relapses (lower relapse rate)	[55]
Decreased risk of overdosing	[46]
Improved pharmacokinetic profile (greater bioavailability, avoidance of first-pass metabolism, good correlation between the dose and plasma concentration, lower and less frequent peak plasma level—reduced side effects)	[47]
Improved patient outcomes and satisfaction	[58]
Regular contact between the patient and mental healthcare team	[8]
DISADVANTAGES	
Slow dose titration, and longer time to obrain steady drug concentration in plasma	[46]
Lower flexibility in adjustement of dose	[45]
Adverse drug effects at the injection site	[56]
Frequent commuting to the hospital or clinic or visits of nurses at home	[55]

**Table 4:** The basic pharmacokinetic characteristics of first- and second-generation antipsychotics available as long-acting injectable medications.

Drug	Excipients	Storage	Plasma Conc. Peak (t_max_)	Protein Binding	Vd	Elimination Half-Life	Therap. Plasma Conc.	References
Haloperidol decanoate	15 mg of benzyl alcohol and up to 1 mL of sesame oil	Store between 15 and 30 °C; do not refrigerate or freeze	3 to 9 days after injection	88–92%	8–21 L/kg	3 weeks	1 to 10 ng/mL	[32,50]
Fluphenazine decanoate	Benzyl alcohol 15 mg/mL and sesame oil	Do not store above 25 °C; do not refrigerate or freeze; protect from light	2.5–16 weeks	>90%		7–10 days	0.15 to 0.5 ng/mL	[32]
Flupentixol decanoate	Vegetable oil	Store between 15 and 25 °C; protect from light	7 days after injection	>99% *	14 L/kg *	21 days	1 to 4 ng/mL	[32,67]
Zuclopenthixol decanoate	Vegetable oil	Store between 15 and 25 °C; protect from light	3–7 days	98–99%	20 L/kg *	19 days	>10 ng/mL	[32,68]
Aripiprazole monohydrate	Carmellose, mannitol, dihydrogen phosphate monohydrate. NaOH, water for injection	Prefilled dual chamber syringe: store below 30 °C; do not freeze; protect from light	4–7 days	99%	4.9 L/kg	29–46 days	ca. 175 ng/mL	[32,69]
Aripiprazole lauroxil	Sulfobutylether β-cyclodextrin (SBECD), tartaric acid, sodium hydroxide, water for injection	Store at room temperature between 20 and 25 °C	41 days	99%	268 L	53–57 days	ca. 200 ng/mL (100–340 ng/mL)	[32,70,71]
Olanzapine pamoate	Carmellose sodium, Mannitol, polysorbate 80, water for injections	Store at room temperature (do not exceed 30 °C)	2–6 days	93%	1100 L for oral dose	ca. 30 days	4.2 to 73.2 ng/mL	[72]
Paliperidone palmitate	Polysorbate 20, polyethylene glycol 4000, citric acid monohydrate, disodium hydrogen phosphate anhydrous, water for injection	Store at room temperature (25 °C)	13 days	74%	390 L	25–49 days	20–60 ng/mL	[73,74]
Risperidone microspheres	Polysorbate 20, carmellose sodium, disodium hydrogen phosphate, citric acid anhydrous, sodium chloride, sodium hydroxide, water for injection	Store in refrigerator between 2 and 8 °C; protect from light	30 days	90%	1–2 L/kg	3–6 days	10–45 nmg/mL	[47,59,75]

Vd—Volume of distribution; *—data given for parent drug (flupentixol, zuclopentixol etc.). Excipients in the drug formulations were listed based on the information collected at https://www.medicines.org.uk/emc/ (accessed on 2 October 2023).

**Table 5:** Example of a specification for a lyophilizate product for sustained release injection [120].

Attribute	Method	Requirements	Function
Appearance	Visual evaluation	Example: white to off-white opaque suspension	Confirming stability
Resuspension time	Visual evaluation	≤0 s	Quality
Identification of API ^1^	XRPD ^2^/ HPLC ^3^/ HPLC PDA ^4^/ mass spectrometry	Presence of certain peaks, absence of certain peaks/compliance of retention time with chromatogram of standard solution/compliance with spectrum of standard solution	Identity
Water content	Volumetric/coulometric Karl Fischer, near-IR ^5^	NMT ^6^ X%	Quality Confirming stability
Particle size distribution (PSD)	Laser diffraction	Dv10 > w, Dv50 x–y and Dv90 < z ^7^	Quality Product performance
Particulate contamination	Visible particles Ph. Eur.^8^ § 2.9.20.	No contamination visible with naked eye	Quality
In vitro release	Apparatus 1 (Basket Apparatus) (USP ^9^); Apparatus 2 (Paddle Apparatus) (USP); Apparatus 4 (Flow-Through Cell) (USP); methods using dialysis membrane; samples taken and measured by HPLC or UV spectroscopy	Initial release: NMT X% Complete release: NLT ^10^ % 3–5 time-points	Quality Product performance Confirming stability
Purity	HPLC	Total and individual related substances	Quality Confirming stability
Assay	HPLC or UV spectroscopy	% of active substance in relation to the declared dose	Strength Confirming stability
Content uniformity	HPLC or UV spectroscopy	acceptance value (AV) ≤ X	Strength
Sterility testing	Membrane filtration method	Sterile	Safety
Test for bacterial endotoxins	LAL test ^11^	NMT XX IU/mg	Safety

^1^. API—Active Pharmaceutical Ingredient. ^2^. XRPD—X-Ray Powder Diffraction. ^3^. HPLC—High-performance liquid chromatography. ^4^. PDA—Photodiode-Array Detection. ^5^. near-IR—near Infrared spectroscopy. ^6^. NMT—not more than. ^7^. Dv10, Dv50, Dv90—Particle size distribution corresponding to the percentages 10%, 50%, and 90% of particles under the reported particle size. ^8^. Ph. Eur.—European Pharmacopoeia. ^9^. USP—United States Pharmacopeia. ^10^. NLT—not less than. ^11^. LAL test—limulus amebocyte lysate test.
